# The Role of Diffusion Tensor Imaging in CNS Tuberculosis

**DOI:** 10.7759/cureus.62998

**Published:** 2024-06-23

**Authors:** Saurya Saurya, Garima Sharma, Brig Sudhir Saxena, Puneet K Gupta

**Affiliations:** 1 Department of Radiodiagnosis, All India Institute of Medical Sciences, Rishikesh, Rishikesh, IND; 2 Department of Microbiology, All India Institute of Medical Sciences, Rishikesh, Rishikesh, IND

**Keywords:** neurologic prognosis, intracranial tuberculoma, • fractional anisotropy (fa), cns tuberculosis, • diffusion tensor imaging (dti)

## Abstract

Background and objective

Tuberculosis (TB), caused by Mycobacterium tuberculosis, remains a significant global health concern, with India being a hotspot for the disease burden. Central nervous system (CNS) tuberculosis, though comprising a smaller proportion of total TB cases, is associated with significant morbidity and mortality. This study aimed to explore the utility of diffusion tensor imaging (DTI) in assessing the microstructural changes in white matter tracts associated with CNS tuberculosis.

Materials and methods

This study was conducted over two years at the All India Institute of Medical Sciences, Rishikesh. We employed a cross-sectional observational design and included patients with definite or highly probable tuberculous meningitis, alongside healthy controls.

Results

Our findings revealed a significant reduction in fractional anisotropy (FA) values in various white matter tracts of patients with CNS tuberculosis compared to healthy individuals. This reduction in FA correlated with the severity of tuberculous meningitis, particularly in the corpus callosum. Additionally, DTI highlighted distinct patterns of white matter involvement around intraparenchymal lesions, suggesting potential implications for clinical outcomes. The study emphasizes the utility of FA values in grading disease severity and prognosticating treatment outcomes in CNS tuberculosis.

Conclusions

Overall, this study provides valuable insights into the microstructural alterations in white matter tracts associated with CNS tuberculosis, highlighting the potential of DTI in early diagnosis, grading disease severity, and monitoring treatment response. We believe these findings will pave the way for further research to optimize the clinical management of this debilitating disease.

## Introduction

Tuberculosis (TB), which is caused by Mycobacterium tuberculosis, is one of the leading causes of death from a single infectious agent (rivaling HIV/AIDS) [1]. Mycobacterium tuberculosis infection has been primarily classified into two main categories: pulmonary and extrapulmonary TB. Even though TB can involve any organ, the common sites of involvement in extrapulmonary TB are the central nervous system (CNS), lymph nodes, genitourinary tract, pleura, abdomen, bones, and joints [2]. CNS TB accounts for 5-10% of all tuberculous infections and is the most devastating form of this disease. It is associated with high morbidity and mortality. Although it can be potentially life-threatening, it is curable if the correct diagnosis is made in the early stages [3].

Imaging modalities play an important role in not only the early and accurate diagnosis of CNS TB but also in identifying its disabling complications. Among the various radiological modalities, MRI is the investigation of choice as it provides greater sensitivity and specificity compared to CT scans. On imaging, leptomeningeal enhancement with basal exudates is the most common appearance of CNS TB. Leptomeningeal enhancement is commonly seen along peri-mesencephalic, interpeduncular prepontine, and supra-sellar cisterns. Less commonly, enhancement may also seen along the sulcal spaces in the cerebral hemisphere and the Sylvian fissure [4]. While dura involvement is usually secondary to leptomeningitis, direct dural seeding has been reported in a few cases [5]. Parenchymal tuberculoma, which is formed by a conglomeration of parenchymal tuberculous granulomas, has a variable appearance on MRI depending upon their stages [6]. Another manifestation of CNS TB is cerebritis, which appears as a gyriform area of T2 hyperintensity and T1 hypointensity. Gyriform enhancement is usually seen in post-contrast images [3].

Vasculitic infarcts are also seen commonly in tuberculous meningitis. Lenticulostriate and thalamic perforating vessels are commonly involved with associated infarcts in their territory. Venous infarct after dural venous thrombosis may occur in tuberculous meningitis [7]. In 17-70% of cases, there is associated involvement of cranial nerves. The involved nerve appears thickened and shows a hyperintense signal on T2WI. The most commonly involved nerves are the third, fourth, and seventh cranial nerves [8]. Various advanced MRI techniques such as magnetization transfer imaging, diffusion tensor imaging (DTI), and proton magnetic resonance spectroscopy aid in better characterization of CNS TB. However, due to non-specific presentation and overlapping features with other CNS infections, a definitive diagnosis of CNS TB may be contested even on MRI.

DTI is based on the Brownian motion of water molecules and can detect the degree and direction of movement of water molecules. The water molecules in body tissue undergo a random translational movement, which is called Brownian. When this motion occurs freely in all directions without any restriction in one direction, it is termed as isotropic. In tissues, this mobility is restricted in a certain direction by the tissue microstructural barriers; hence, the diffusion is not free in all directions and is termed anisotropic. DTI measures the magnitude and direction of this anisotropic diffusion. Any structure being imaged is broken down into small three-dimensional structures called voxels. The integrity of a linear structure, like a white matter tract, is evaluated by measuring serially the direction as well as the speed of diffusion in adjacent voxels. Alteration in the microstructure of these tracts due to any pathology affecting them may be reflected as a decreased magnitude of diffusion or altered direction of diffusion in these tracts, which can be evaluated using DTI. Thus, DTI can pick up early changes in the white matter tract, much before the change becomes apparent on conventional MRI. DTI is known to provide details about tissue organization and structure which is beyond the scope and resolution of conventional MRI [9].

The role of DTI has been extensively studied in the evaluation of ischemic brain injuries, demyelination, traumatic brain injuries, stroke, infections, inflammation, and edema. Its superiority over conventional MRI techniques has also been reported in several studies where early diagnosis and better microstructural changes could be analyzed using DTI. The application of DTI in CNS TB may help explore and provide evidence of microstructural white matter changes, thereby enabling early diagnosis and better evaluation of the extension of the disease. To the best of our knowledge, there are very few studies evaluating the role of DTI in CNS TB.

## Materials and methods

For this study, we recruited patients from the outpatient and inpatient departments of a tertiary care institute. Informed written consent was obtained from patients and healthy volunteers. Detailed history, examination findings, and biochemical/laboratory investigations like CSF analysis were recorded for all included patients in a predesigned format. The severity of tuberculous meningitis was graded using the British Medical Research Council Criteria as Stage I, Stage II, and Stage III as follows: Stage I (early): “If only nonspecific signs and symptoms and signs are present in the absence of any neurological deficit and clouding of consciousness.” Stage II (intermediate): “in the presence of any of the following: signs of meningeal irritation, minor neurological deficit such as the cranial nerve involvement and or presence of lethargy or behavioral changes." Stage III (late): “in the presence of any of the following: stupor or coma, seizure or abnormal body movement and/or neurological deficit such as limb weakness”.

Healthy volunteers and patients with a definitive diagnosis of tuberculous meningitis according to the Lancet consensus scoring system or a diagnosis of highly probable tubercular meningitis based on criteria proposed by Ahuja et al. underwent an MRI of the brain on a 3-T MR Scanner (GE HealthCare, Chicago, IL) by using a dedicated CP array head coils. In healthy volunteers, non-contrast MR scanning of the brain was performed and the following sequences were acquired: T1WI, T2WI, FLAIR, and SWI images in axial/coronal/sagittal planes. Multidirectional diffusion (30 direction) weighted images were obtained to acquire DTI images for the whole brain. In CNS TB patients, the following sequences were acquired: T1W, T2W, FLAIR, and SWI images in axial/coronal/sagittal planes; multidirectional diffusion-weighted sequences to acquire DTI images for the whole brain; and post-contrast images after intravenous injection of gadolinium contrast agent at a dose of 0.1 mmol/kg followed by 20 ml saline chase using a 3D dynamic fat saturated T1W technique. The images acquired were evaluated for MRI features of CNS TB.

All the data were transferred to a workstation. Post-processing of raw DTI data was done using READY View on the Advantage 4.7 workstation (GE HealthCare). Color-coded FA maps and grayscale ADC maps were generated. Region of interest circles of uniform size were drawn and DTI parameters like fractional anisotropy (FA) and Apparent diffusion coefficient (ADC) were recorded in the following regions - in healthy individuals: bilateral Inferior longitudinal fasciculus, bilateral superior longitudinal fasciculus, bilateral cingulum, bilateral inferior frontal occipital fasciculus, bilateral superior fronto-occipital fasciculus, bilateral uncinate fasciculus, bilateral forceps major, anterior limb, genu and posterior limb of the bilateral internal capsule, the body of corpus callosum, and bilateral corticospinal tract; in TB meningitis: bilateral inferior longitudinal fasciculus, bilateral superior longitudinal fasciculus, bilateral cingulum, bilateral inferior fronto-occipital fasciculus, bilateral superior fronto-occipital fasciculus, bilateral uncinate fasciculus, bilateral forceps major, anterior limb, genu and posterior limb of bilateral internal capsule, body of corpus callosum, and bilateral corticospinal tract; in patients with focal parenchymal lesions: at the center of the lesion and in the perilesional white matter. Mirror ROI in the contralateral cerebral hemisphere was also drawn. Comparative analysis of DTI findings between patients with CNS TB and healthy volunteers was done and results were analyzed. Also, the DTI findings in different grades of TB meningitis were compared.

Inclusion and exclusion criteria

The inclusion criteria were as follows: Age ≥13 years; definitive diagnosis of tuberculosis meningitis based on the Lancet consensus scoring system or diagnosis of highly probable tuberculous meningitis as per the criteria established by Ahuja et al. (Appendices 1); patient willing to give written informed consent. The exclusion criteria were as follows: patients with contraindication to MRI, i.e., patients with MRI-incompatible implant or claustrophobia; patients with contraindication to intravenous contrast, such as contrast allergy, and deranged renal function; seropositivity for HIV; pregnancy; patients with other co-existing disease of CNS at present or in the past.

**Figure 1:**
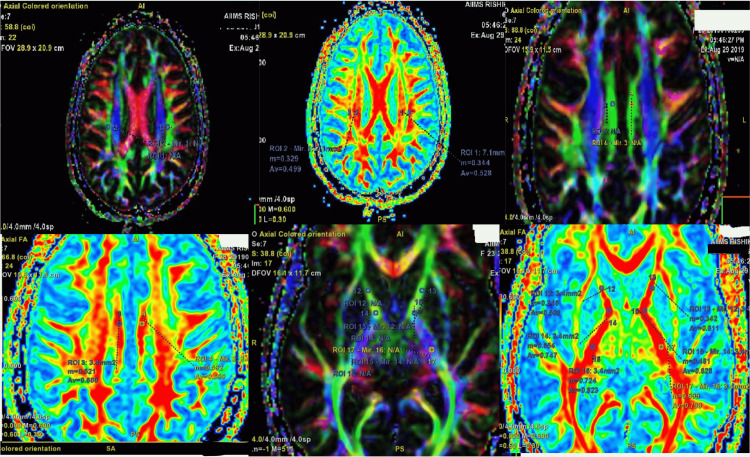
Representative images showing fractional anisotropy values being measured in various white matter tracts Figures sourced from the authors' collection of cases and from the study sample

Mean values of FA and mean diffusion coefficient in CNS TB patients in the regions described were compared with the FA and mean diffusion coefficient in the corresponding areas of the brain in healthy volunteers (Figure 1).

Data were imported into an MS Excel sheet and analyzed using SPSS Statistics version 17 software (IBM Corp., Armonk, NY). The difference in mean FA value between CNS TB patients and healthy volunteers was calculated using the student's t-test for normally distributed data or the Mann-Whitney U test for non-normal data. A p-value <0.05 was considered statistically significant.

## Results

A total of 43 cases were initially recruited for the study out of which 13 patients were excluded: 11 of them were too critical to be shifted for MRI or expired before the scheduled MRI; two patients were excluded due to excessive motion artifacts in imaging. Ultimately, a total of 30 patients were included in the study: nine were definite tuberculous meningitis patients and 22 were highly probable tuberculous meningitis; 30 healthy age and sex-matched controls were also included. Statistically significant reduced FA values were seen in cases compared to control in bilateral inferior longitudinal fasciculus, bilateral superior longitudinal fasciculus, bilateral cingulum, bilateral inferior fronto-occipital fasciculus, bilateral inferior fronto-occipital fasciculus, bilateral uncinate fasciculus, anterior limb of bilateral internal capsules, and corpus callosum body (Table 1).

**Table 1:** Summary of results ^*^Significant at p<0.05; ^1^Fisher's exact test; ^2^chi-squared test; ^3^Wilcoxon-Mann-Whitney U test; ^4^t-test ADC: apparent diffusion coefficient; FA: fractional anisotropy; SD: standard deviation

All Parameters	Group		P-value
	Cases (n = 30)	Controls (n = 30)	
Age, n (%)			0.9621
≤20 Years	7 (23.3%)	6 (20.0%)	
21-30 Years	11 (36.7%)	9 (30.0%)	
31-40 Years	5 (16.7%)	6 (20.0%)	
41-50 Years	2 (6.7%)	4 (13.3%)	
51-60 Years	3 (10.0%)	4 (13.3%)	
61-70 Years	1 (3.3%)	1 (3.3%)	
71-80 Years	1 (3.3%)	0 (0.0%)	
Gender, n (%)			1.0002
Male	12 (40.0%)	12 (40.0%)	
Female	18 (60.0%)	18 (60.0%)	
FA: Inferior Longitudinal Fasciculus (Right)^*^, mean ± SD	0.44 ± 0.09	0.54 ± 0.07	<0.001^3^
Fa: Inferior Longitudinal Fasciculus (Left)^*^, mean ± SD	0.39 ± 0.10	0.52 ± 0.05	<0.001^3^
ADC: Inferior Longitudinal Fasciculus (Right)^*^, mean ± SD	2979.20 ± 466.14	2695.33 ± 340.61	0.0094
ADC: Inferior Longitudinal Fasciculus (Left)^*^, mean ± SD	3233.03 ± 457.75	2973.10 ± 337.47	0.0154
FA: Superior Longitudinal Fasciculus (Right)^*^, mean ± SD	0.46 ± 0.12	0.58 ± 0.06	<0.001^4^
FA: Superior Longitudinal Fasciculus (Left)^*^, mean ± SD	0.49 ± 0.09	0.56 ± 0.04	<0.001^4^
ADC: Superior Longitudinal Fasciculus (Right), mean ± SD	2912.47 ± 940.99	2699.13 ± 261.04	0.3553
ADC: Superior Longitudinal Fasciculus (Left), mean ± SD	2894.60 ± 383.40	2915.87 ± 291.82	0.8104
FA: Cingulum (Right)^*^, mean ± SD	0.53 ± 0.13	0.61 ± 0.08	0.0103
FA: Cingulum (Left)^*^, mean ± SD	0.53 ± 0.11	0.63 ± 0.08	<0.001^4^
ADC: Cingulum (Right), mean ± SD	2814.97 ± 512.93	2750.07 ± 343.73	0.9183
ADC: Cingulum (Left), mean ± SD	2837.73 ± 507.45	2802.77 ± 302.45	0.7474
FA: Inferior Fronto-occipital Fasciculus (Right)^*^, mean ± SD	0.44 ± 0.12	0.57 ± 0.07	<0.001^4^
FA: Inferior Fronto-occipital Fasciculus (Left)^*^, mean ± SD	0.43 ± 0.11	0.55 ± 0.07	<0.001^4^
ADC: Inferior Fronto-occipital Fasciculus (Right), mean ± SD	2722.37 ± 582.11	2477.80 ± 565.38	0.3263
ADC: Inferior Fronto-occipital Fasciculus (Left), mean ± SD	2630.30 ± 488.20	2416.93 ± 411.68	0.1563
FA: Superior Fronto-occipital Fasciculus (Right)^*^, mean ± SD	0.38 ± 0.13	0.50 ± 0.06	<0.001^4^
FA: Superior Fronto-occipital Fasciculus (Left)^*^, mean ± SD	0.39 ± 0.12	0.51 ± 0.05	<0.001^3^
ADC: Superior Fronto-occipital Fasciculus (Right)^*^, mean ± SD	3489.27 ± 1331.30	2700.20 ± 304.47	0.0033
ADC: Superior Fronto-occipital Fasciculus (Left), mean ± SD	3703.37 ± 1711.11	2843.07 ± 332.56	0.1433
FA: Uncinate Fasciculus (Right)^*^, mean ± SD	0.47 ± 0.10	0.59 ± 0.06	<0.001^4^
FA: Uncinate Fasciculus (Left)^*^, mean ± SD	0.43 ± 0.09	0.57 ± 0.07	<0.001^4^
ADC: Uncinate Fasciculus (Right), mean ± SD	2752.70 ± 489.64	2600.97 ± 324.35	0.1634
ADC: Uncinate Fasciculus (Left), mean ± SD	2697.73 ± 592.07	2498.47 ± 361.60	0.1023
FA: Forceps Major (Right), mean ± SD	0.80 ± 0.10	0.84 ± 0.09	0.1244
FA: Forceps Major (Left), mean ± SD	0.80 ± 0.13	0.84 ± 0.07	0.4333
ADC: Forceps Major (Right), mean ± SD	2769.33 ± 688.56	2551.63 ± 447.13	0.1503
ADC: Forceps Major (Left), mean ± SD	2720.27 ± 462.51	2632.07 ± 350.26	0.4094
FA: Anterior Limb of Internal Capsule (Right)^*^, mean ± SD	0.50 ± 0.11	0.58 ± 0.07	0.0014
FA: Genu of Internal Capsule (Right), mean ± SD	0.66 ± 0.09	0.67 ± 0.07	0.6874
FA: Posterior Limb of Internal Capsule (Right), mean ± SD	0.74 ± 0.08	0.72 ± 0.08	0.1413
FA: Anterior Limb of Internal Capsule (Left)^*^, mean ± SD	0.48 ± 0.12	0.63 ± 0.09	<0.001^4^
FA: Genu of Internal Capsule (Left), mean ± SD	0.64 ± 0.07	0.67 ± 0.06	0.0744
FA: Posterior Limb of Internal Capsule (Left), mean ± SD	0.70 ± 0.12	0.72 ± 0.05	0.9763
ADC: Anterior Limb of Internal Capsule (Right), mean ± SD	2753.27 ± 675.36	2512.50 ± 412.85	0.1024
ADC: Genu of Internal Capsule (Right), mean ± SD	2594.33 ± 412.70	2446.07 ± 275.51	0.1084
ADC: Posterior Limb of Internal Capsule (Right)^*^, mean ± SD	2905.27 ± 329.59	2719.17 ± 354.35	0.0404
ADC: Anterior Limb of Internal Capsule (Left)^*^, mean ± SD	2705.77 ± 577.90	2440.17 ± 388.38	0.0424
ADC: Genu of Internal Capsule (Left), mean ± SD	2690.37 ± 682.23	2514.40 ± 330.70	0.1933
ADC: Posterior Limb of Internal Capsule (Left)^*^, mean ± SD	3263.37 ± 444.08	3003.33 ± 469.60	0.0324
FA: Corpus Callosum Body^*^, mean ± SD	0.48 ± 0.14	0.63 ± 0.06	<0.001^4^
ADC: Corpus Callosum Body^*^, mean ± SD	3964.80 ± 1057.24	3150.00 ± 398.53	<0.001^4^
FA: Corticospinal Tract (Right), mean ± SD	0.64 ± 0.08	0.64 ± 0.07	0.8344
FA: Corticospinal Tract (Left)^*^, mean ± SD	0.63 ± 0.06	0.67 ± 0.07	0.0214
ADC: Corticospinal Tract (Right), mean ± SD	2892.17 ± 416.28	2854.23 ± 427.76	0.7294
ADC: Corticospinal Tract (Left), mean ± SD	2964.20 ± 460.13	2908.27 ± 563.31	0.3913

**Figure 2:**
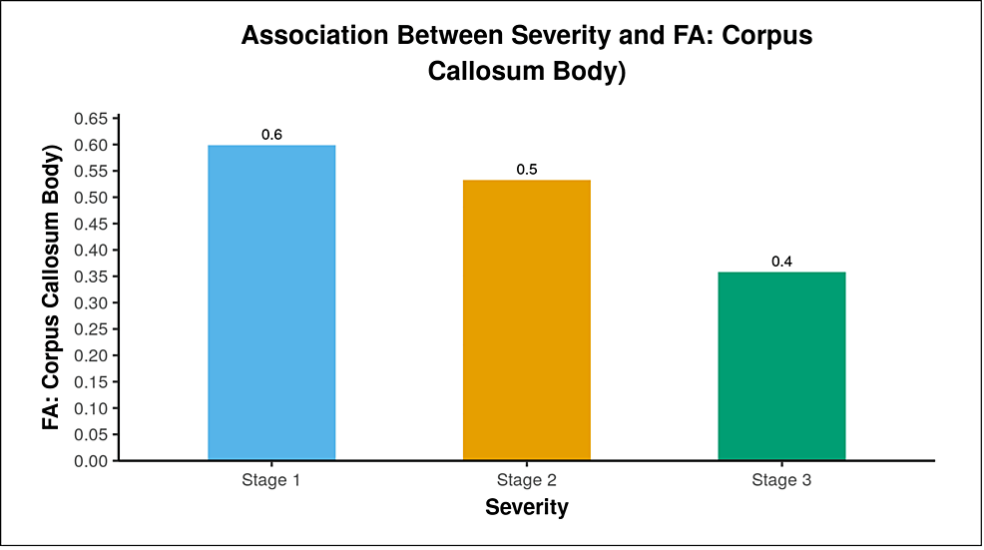
Bar graph showing means of FA: corpus callosum body, in the three groups (Stage I, Stage II, and Stage III) FA: fractional anisotropy

Grades of the severity of tuberculous meningitis (according to British Medical Research Council criteria) were compared with FA values in various white matter tracts. Reduction in FA values in the corpus callosum statistically correlated with worsening in the grade of tuberculous meningitis (Figure 2).

**Figure 3:**
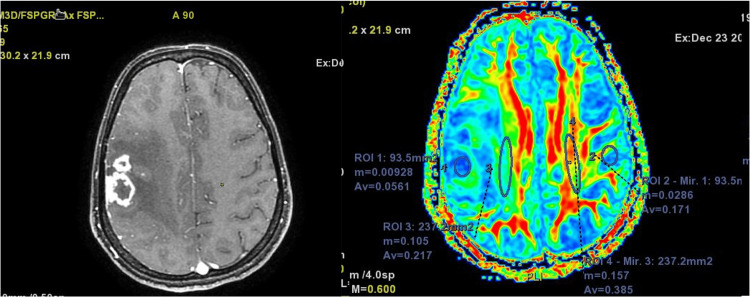
Representative images: post-contrast T1WI, (left) FA map (right) DTI color map; FA value in the center of lesion nears zero. The central part of the lesion has almost isotropic diffusion. The perilesional white matter shows reduced FA Figures sourced from the authors' collection of cases and from the study sample DTI: diffusion tensor imaging; FA: fractional anisotropy

The present study showed a significant reduction in anisotropy in white matter adjacent to parenchymal lesions compared to the mirror contra-lateral normal side (Table 2). Fractional anisotropy values were very low in the center of the lesion, with values approaching zero in some lesions (Figure 3).

**Table 2:** Comparison between FA of parenchymal lesion and mirror contralateral normal brain parenchyma FA: fractional anisotropy; IQR: interquartile range; SD: standard deviation

Variable	DTI Parameters			Wilcoxon Test	
	Mean (SD)	Median (IQR)	Range	V	P-value
Lesion FA	0.13 (0.08)	0.10 (0.04)	0.06-0.36		
Mirror FA	0.30 (0.13)	0.25 (0.16)	0.17-0.60	0	<0.001
Absolute Difference	0.17 (0.09)	0.13 (0.13)	0.08-0.39		
Percentage Difference	166.2% (142.7)	125.6% (89.5)	36%-616%		

## Discussion

We observed a statistically significant reduction in FA values in the majority of the white matter tracts in cases compared to healthy age-matched controls (Table 1). The tracts showing reduced FA were as follows: bilateral inferior longitudinal fasciculus, bilateral superior longitudinal fasciculus, bilateral cingulum, bilateral inferior fronto-occipital fasciculus, bilateral inferior fronto-occipital fasciculus, bilateral uncinate fasciculus, anterior limb of bilateral internal capsules, and corpus callosum body. Our findings are consistent with previous studies evaluating changes in FA values in the white matter tract in patients with meningitis. A study by Lin et al. (2013) involving patients with chronic meningitis reported a similar decrease in FA of various white matter tracts [10]. The decreased anisotropy in the white matter tract reflects the microstructural damage to axons in tuberculous meningitis [11], which can be attributed to various factors, like the inflammatory milieu in meningitis, the vasculitis involving the small and medium-sized vessels, hydrocephalus compressing upon the white matter tracts, and the tuberculous parenchymal lesions, and edema surrounding them.

Primarily, tuberculous bacilli infect the microglia cells in the brain. Upon being infected, these cells are activated and synthesize a variety of inflammatory mediators. Through the activation of the NADPH oxidase pathway, reactive oxygen species are generated. These reactive oxygen species may result in direct damage to white matter tracts by causing oxidation of the membrane lipids. TNF alpha and other mediators of inflammation are synthesized, which further leads to an increase in blood-brain barrier permeability. This leads to a further increased influx of inflammatory mediators. Matrix metalloproteinase, an enzyme that causes proteolysis of extracellular matrix, has been found in increased concentration around tuberculous parenchymal lesions. Thus, the inflammatory milieu in TB meningitis may well be associated with white matter microstructural damage [11,12].

The major vessels supplying the brain parenchyma are bathed in exudates, primarily in the region of basal cisterns, and Sylvian fissures. This leads to a contiguous extension of inflammation from the subarachnoid space to the adventitia and subsequently intima of vessels, leading to pan arteritis. In cases with chronic meningitis and those with incomplete treatment of meningitis, there is intimal proliferation, leading to the thickening of arteries and their occlusion. In small arteries and arterioles, vessel wall necrosis is associated with TB meningitis. These vascular pathologies, especially those involving the small arteries and arterioles, may be responsible for ischemic damage to white matter and can explain the diffuse decreased anisotropy in TB meningitis [7,11]. Hydrocephalus is very common in TB meningitis. The mechanical pressure due to hydrocephalus can cause periventricular white matter injury in TB meningitis [10]. In our study, the white matter tract adjacent to the lateral ventricle and the bilateral superior fronto-occipital fasciculus were some of the most severely affected white matter tracts, showing the least mean FA value among the various white matter tracts evaluated. This can be attributed to the additional effect of compression of the tracts by hydrocephalus, a very common complication of TB meningitis.

In the present study, even though FA values were seen to decrease in the majority of the white matter tracts, significant changes in ADC value were not observed in most of the white matter tracts (Table 1). Similar findings were observed by Malik et al. in periventricular white matter tracts in cases with meningitis. On comparing meningitis patients having normal outcomes with those having abnormal outcomes, they observed a decrease in FA values in white matter tracts in patients with abnormal outcomes; however, no increase in mean diffusivity was observed. In addition, when they compared controls with patients having meningitis (normal outcome), they found a decrease in FA value with no increase in mean diffusivity. These findings could be attributed to the pseudo-normalization of ADC weeks after the insult [13]. Moreover, ADC is a scalar measurement and measures diffusion in all directions, whereas the directional diffusion in white matter tracts is better characterized by FA [14].

We compared grades of severity of tuberculous meningitis (according to the British Medical Research Council criteria) with FA values in various white matter tracts (Figure 2). Reduction in FA values in the corpus callosum statistically correlated with a worsened grade of TB meningitis. This could be attributed to the fact that the corpus callosum is the primary commissural region of the brain connecting both the cerebral hemispheres. It is the largest white matter bundle [15], and any damage to it is very likely to present with worsened severity of tuberculous meningitis. Lin et al. correlated FA values of various white matter tracts with neuropsychological parameters like attention, speech and language execution, and amnesic and visuoconstruction functions in patients with chronic meningitis [10]. There was a correlation between reduced anisotropy and poor attention, execution, and visuoconstruction functions. In the present study, however, neuropsychological parameters were not evaluated; rather, the grading of the severity of patients' conditions was done using the British Medical Council research criteria, which do not incorporate much of the neuropsychological status of patients.

In the present study, FA values were very low in the center of the lesion, with values approaching zero in some lesions (Figure 3). This is attributed to the necrosis in the central region of the parenchymal granulomas. Due to necrosis and liquefaction, the brain tissue is replaced by fluid-like material, which has near isotropic diffusion. The relatively lower reduction in FA values in many of the lesions may be attributed to the various stages of the tuberculous parenchymal granulomas. The near-zero FA could be seen in caseating granulomas with central liquefaction, whereas relatively less reduction in FA could be attributed to caseating granulomas without central necrosis and noncaseating granulomas [6,16,17].

Even though the mean ADC value in lesions was reduced as compared to the mirror contralateral side in this study, the reduction was not statistically significant. This could be attributed to two different processes occurring simultaneously in parenchymal lesions, which have opposite effects on ADC. The process of liquefaction increases the diffusivity and ADC whereas the process of inflammation and cellular infiltration increases the cellularity and reduces the diffusivity. To better understand this, we need to look at the variation in histology of granulomas depending upon the stage of granulomas. Initially, granuloma consists of specialized macrophages called epithelioid cells surrounding a necrotic area. The involved brain parenchyma can undergo caseation, liquefaction, or calcification. Some granulomas may not have necrotic areas at all and may have high cellularity with plenty of macrophages and lymphocytes. This variation in the histology of granulomas is due to adaptive changes in virulence factors of different tuberculous bacilli in response to host immunity [16,17,18].

We found a significant reduction in anisotropy in white matter adjacent to parenchymal lesions compared to the mirror contra-lateral normal side (Table 2), which is consistent with a previous similar study done by Gupta et al. among brain tuberculoma patients. They also observed decreased anisotropy in perilesional areas in brain tuberculoma [17]. The reduced FA values in the present study were seen in both the areas with visible edema on T2/FLAIR images as well as in the normal-appearing areas on T2/FLAIR images. As the increase in interstitial water is associated with decreased anisotropy in various studies, perilesional edema is one of the reasons for the reduced FA [19]. Another reason for decreased FA could be the expression of matrix metalloproteinases and other proteases in the lesion causing destruction of the extracellular matrix and microstructural damage to adjacent white matter. In the study by Gupta et al., the decrease in perilesional FA normalized with a decrease in perilesional edema on a follow-up scan, further reinstating the fact that the major contribution to decreased FA in perilesional white matter is due to edema [17].

The brain parenchymal edema is broadly classified into cytotoxic and vasogenic edema. The cytotoxic edema is associated with reduced ADC values whereas the vasogenic edema is associated with increased ADC. Vasogenic edema is caused by the disruption of the endothelial junction, with increased fluid in between the multiple parallel axon tracts. In the present study, the mean ADC value in the perilesional white matter was also observed to be higher than the contralateral mirror brain parenchyma. This could also be attributed to the surrounding vasogenic edema [20]. On color-coded DTI maps, there are four patterns in which the white matter tract can be affected by a lesion. The white matter tract can be either deviated, infiltrated, edematous, or destroyed by the lesion. Displaced white matter tracts have normal anisotropy; however, their continuity, color hue, and color intensity remain the same on color-coded DTI maps. These tracts may show mildly decreased FA values.

Edematous tracts on color-coded diffusion tensor images show normal color hue and continuity, suggesting that the direction of anisotropy as well as the continuity of white matter tracts are well maintained. Such tracts, however, show a significant decrease in FA values. White matter tracts can show complete disruption; in such cases, the tracts will show abnormal color as well as decreased FA. This pattern has been attributed to tumor infiltration into the white matter tract. The last pattern involves a destroyed tract where the fiber cannot be identified on a color-coded map. The FA value in such cases becomes near to isotropy [21]. In our case, the perilesional white matter showed maintained orientation with normal color; however, the brightness of the color was reduced, indicating the edematous pattern of involvement. The center of lesions, which were large enough to be characterized, showed disruption of the white matter tract with near complete isotropy. Color-coded DTI map showed the destruction of fibers in the center of the lesion, while the perilesional white matter showed an edematous pattern.

## Conclusions

We believe this study has accomplished its aims and objectives in defining the role of DTI in CNS tuberculosis. The study has conclusively revealed reduced FA in the majority of white matter tracts bilaterally in patients with CNS tuberculosis as compared to healthy controls. It may be hypothesized that the cognition decline and other symptoms of the patient, which could not be explained by meningeal inflammation or parenchymal brain lesions alone, are attributable to the reduced FA in white matter tracts, which aligns with previous similar studies. There are changes in FA values that correlate with the severity of meningitis as graded by the British Medical Research Council criteria in corpus callosum.

We observed reduced FA with near complete isotropy and destruction of fibers in the center of intraparenchymal ring-enhancing lesion compared to normal contralateral brain parenchyma, whereas the perilesional white matter showed reduced FA with increased ADC. The continuity, orientation, and direction of perilesional white matter tracts were well maintained. These findings are consistent with the results of a previous study on CNS tuberculomas. It may be hypothesized that the improvement in the patient’s clinical condition on treatment is attributable to this pattern of involvement of the perilesional white matter tract, where the white matter tracts are intact with their orientation and direction well maintained. The destruction of fibers in the center may be attributed to residual deficits that remain in some patients even after treatment.

## Appendices

**Table 3:** The Lancet Consensus Criteria for the Diagnosis of Definite Tuberculous Meningitis This table was created by the authors based on the Lancet criteria [22] CSF: cerebrospinal fluid

Clinical Entry Criteria	Criteria A	Criteria B
Meningitis including one or more of the following: headache, irritability, vomiting, fever, neck stiffness, convulsions, focal neurological deficits, altered consciousness, or lethargy	Clinical entry criteria + acid-fast bacilli seen in the CSF; Mycobacterium tuberculosis cultured from the CSF; or a CSF-positive commercial nucleic acid amplification test	Acid-fast bacilli seen in the context of histological changes consistent with tuberculosis in the brain or spinal cord with suggestive symptoms or signs and CSF changes, or visible meningitis (on autopsy)

**Table 4:** The Ahuja Criteria for the Diagnosis of Highly Probable Tuberculous Meningitis This table was created by the authors based on the Ahuja criteria [23] CSF: cerebrospinal fluid

A. Clinical	B. CSF	C. Radiological	D. Extraneural tuberculosis
Fever and headache lasting for more than 14 days (mandatory). Vomiting, alteration of sensorium, or focal deficit (optional)	Pleocytosis with more than 20 cells, predominantly (greater than 60%) lymphocytes, protein greater than 100 mg%, sugars less than 60% of corresponding blood sugars. Negative India ink studies and cytology for malignant cells (in relevant situations)	Imaging studies of the head showing 2 or more of the following: 1. exudates in basal cisterns or Sylvian fissures; 2. hydrocephalus; 3. infarcts; 4. gyral enhancement	Active tuberculosis of the lungs, gastrointestinal tract, urogenital tract, lymph nodes, skeletal system, or skin as evidenced by appropriate radiological or microbiological tests or by the presence of caseation necrosis on histopathological examination
Highly Probable TB meningitis: clinical criteria A + all 3 of (B), (c), and (D)			
